# Human cerebral organoids: the ethical stance of scientists

**DOI:** 10.1186/s13287-023-03291-x

**Published:** 2023-04-01

**Authors:** Andrea Lavazza, Alice Andrea Chinaia

**Affiliations:** 1 Department of Brain and Behavioral Sciences, University of Pavia, Piazza Botta 11, 27100 Pavia, Italy; 2 MoMiLab, IMT School for Advanced Studies Lucca, Piazza S. Francesco 19, 55100 Lucca, Italy

**Keywords:** Neuroethics, Human cerebral organoids, Qualitative analysis, Consciousness, Moral status, Mini-brains, Informed consent

## Abstract

**Background:**

Human cerebral organoids (HCOs) offer unprecedented opportunities to study the human brain in vitro*,* but they also raise important ethical concerns. Here we report the first systematic analysis of scientists’ stance within the ethical debate.

**Method:**

Twenty-one in-depth semi-structured interviews were analyzed through a constant comparative method to highlight how the ethical concerns filter in the laboratory.

**Results:**

The results suggest that the potential emergence of consciousness is not yet seen with concern. However, there are some features of HCO research that need to be better accounted for. Communication to the public, the use of terms such as “mini-brains”, and informed consent appear to be the most pressing concerns of the scientific community. Nonetheless, respondents generally showed a positive attitude toward the ethical discussion, recognizing its value and the necessity of constant ethical scrutiny over scientific advancements.

**Conclusions:**

This research paves the way for a better-informed dialogue between scientists and ethicists, highlighting the issues to be addressed whenever scholars of different backgrounds and interests meet.

**Supplementary Information:**

The online version contains supplementary material available at 10.1186/s13287-023-03291-x.

## Background

Human cerebral organoids (HCOs) are three-dimensional models grown in vitro either starting from embryonic (ES) or induced pluripotent (iPSC) stem cells, mimicking the developmental process and organization of the developing human brain. Since the first breakthrough paper [13], HCOs have continued to show their potentialities, but have also sparked interest and raised several issues. The fast pace of the research and some features of cerebral organoids—such as their potential capacity to receive sensory inputs [6], send functional outputs [7], or present synchronized electrical activity [18, 20] and exhibit a form of learning [8]—have increased ethical concerns. This has given rise to controversy about the possibility of cultivating conscious brain organoids and the need for new rules for this area of research.

Several scholars have begun to consider the implications of the potential emergence of sentience (the basic ability to feel pain and pleasure) or even consciousness (more complex subjective phenomenal experiences) in HCOs [5, 12, 14, 15, 19]. Given the difficulty of detecting the presence of consciousness in HCOs, the precautionary principle—treating as conscious any cerebral organoids that have the minimal structures deemed necessary for the emergence of consciousness in humans—has recently been proposed as the default ethical position [1, 16]. Based on the potential presence of consciousness, consideration has then been given to whether and what moral status should be granted to HCOs.

It should be noted that not all ethicists agree with this view, and some prominent authors in the field have no concerns related to the sentience/consciousness of HCOs, e.g., [10]. A recent recommendation from the National Academies of Science (2021) did not identify any need for new rules in this field. But the academic discussion has dripped into the public debate. The European Union has recently funded a few projects to assess the ethical aspects of organoid research and the attitudes of lay people, patients, and other stakeholders. Ethical issues may also arise regarding the donation of biological samples: these include the possible uses of the organoids, as well as the donors’ consent and privacy. In addition, other relevant questions are related to the creation of chimeras with HCOs and their connection to digital devices to create new types of biological hybrids [4, 8]. Recently, the discussion has entered the laboratory, and, besides neuroethicists, researchers have also started raising questions [11].

To date, the ethical dimension of HCOs has been investigated from the perspectives of patients. Three different studies, two in the Netherlands [2, 9] and one in the US [3], have qualitatively analyzed the opinions of patients from various disease populations and cultural backgrounds over the ethical issues revolving around organoid research in general—and HCOs in particular.

However, the perspectives of the members of the scientific community have remained marginal. Given the relevance of the ethical considerations and the importance of communicating science to the lay public, it is pivotal to address this gap in the literature. The present work was approved by the Ethical Board of the Department of Brain and Behavioral Sciences of the University of Pavia (IT) (No. 77—2021).

## Method

In accordance with other research, we opted for the usage of in-depth semi-structured interviews, targeting researchers working with HCOs on a daily basis. A total of 21 in-depth semi-structured interviews were carried out remotely, on Zoom, between December 2020 and February 2022. The interviews were conducted in English and lasted for an average of 33 min.

We prepared a list of questions based on the existing literature, considering the following themes: personal experience with HCOs and ethical issues, perspectives on consciousness, moral status, human/non-human chimeras, current and future guidelines, research protocols, and elements of good governance. Given the relative flexibility of a semi-structured approach, the list worked as a canvas, meaning that the order and number of questions were not strictly followed. We preferred, instead, to follow the participants’ train of thought.

Participants (see Table 1 for the demographic specifications) were contacted via email. Invitation emails were sent to principal investigators (PI), asking them, when possible, to also indicate a suitable team member. The sampling process was halted after 21 interviews, when we felt that scientists’ answers repeated or overlapped in a meaningful way.

**Table 1:** Interviewees’ demographic characteristics (*n* = 21)

Demographic information	%
*Gender*	
Male	42.9% [*n* = 9]
Female	57.1% [*n* = 12]
*Age*	
20–29	9.5% [*n* = 2]
30–39	28.6% [*n* = 6]
40–49	47.6% [*n* = 10]
50–59	4.8% [*n* = 1]
60–69	9.5% [*n* = 2]
*Area of expertise*	
Biology and related fields	80.9% [*n* = 17]
Bioengineering	9.5% [*n* = 2]
Neurology	4.8% [*n* = 1]
Neurosurgery	4.8% [*n* = 1]
*Years working with HCOs*	
2 years	9.5% [*n* = 2]
3 years	14.3% [*n* = 3]
4 years	28.6% [*n* = 6]
5 years	4.8% [*n* = 1]
6 years	33.3% [*n* = 7]
> 6 years	9.5% [*n* = 2]

*Note*. “Biology and related fields” includes the following scientific areas of expertise: biology, developmental and cellular biology, developmental neurobiology, molecular neuroscience, neurobiology, neuroscience, cell biology, stem cell biology, and interdisciplinary biology

Before the interview, all respondents were asked to fill in a Google Form sheet, in order to provide both personal information and their agreement to the usage of their personal data in accordance with the EU Legislation 2016/679 – GDPR. All participants considered were guaranteed absolute confidentiality.

All interviews were transcribed manually and checked against the recordings. The transcripts were analyzed through a constant comparative method without any software.

## Results

### Theme 1: on consciousness and moral status

Skepticism on the possibility that current HCOs can develop consciousness was a shared theme. Three different reasons were brought in support of this stance, namely, (i) lack of size and complexity, (ii) lack of interaction with the environment, and iii) lack of a precise definition of the concepts of pain, consciousness, as well as validated measurements that could help determine them. As a participant noted, one may argue that “consciousness is fraught, the definition is very unclear. Does it mean you are aware, or does it mean that you have mental thoughts, that you have a mental activity, that you can reason, that you can respond to questions? What does it mean?” (P8).

Respondents’ answers on the issue of consciousness seem to correlate with their views on the moral status of organoids, as well as their personal ethical perspectives on their usage in research, both alone and in combination with animal experimentation. Generally, interviewees agreed that HCOs, given their current developmental level, are in no way ethically different compared to other tissue products used in research: they are important as they are human material, not because they entail any kind of intrinsic property. Participants also agreed on having never experienced any personal ethical concerns about the usage of HCOs or about performing disruptive experiments on them; in addition, there is a widespread lack of concern over chimeric animals and the possibility of their “humanization”, which is deemed to be impossible at least at the current stage of research.

While a group of participants strongly suggested that consciousness in HCOs is not only a remote possibility, but rather something that will never be achieved, others highlighted that even if consciousness—or pain—were to be somehow measured in HCOs, this would not necessarily be of moral relevance: the kind of sentience that HCOs could (maybe) reach would still be comparable to that of flies or grasshoppers. It should be noted, however, that a third group, despite being skeptical of the possibility of conscious HCOs, argued that *if* they became sentient in a way that we can detect, that *would* have repercussions on how HCOs are handled in research, in terms of both guidelines and engraftment into animal models.

### Theme 2: personal experience with HCOs

Many respondents attempted to provide a definition of HCOs. The list of terms used includes: “blobs”, “bunch of cells”, and “clumps of cells”. In general, interviewees stressed the idea that HCOs, despite them being sometimes called “mini-brains”, are not little brains grown in a dish. In this vein, it is worth noting that there is disagreement among researchers on whether the usage of terms such as “mini-brains” should be maintained, or if it would be preferable to stick to a more scientific vocabulary, despite the need to communicate to the general public. On the one hand, there is a need to make research appealing, accounting for the fact that the public is not familiar with the scientific vocabulary and could be misled by it. On the other hand, some argued that using terms like “mini-brains” is a call for trouble, because it may generate the false belief that HCOs are similar to human brains, except in size, which they are not. Leaning toward expressions like “human cerebral organoids”, instead, might be less engaging but more beneficial. It should be considered, at this point, that “mini-brains” is not the only contentious term in the field: terms such as “consciousness” and “chimeras” can also be controversial and lead to possible misconceptions, especially when communicating to a lay audience.

**Fig. 1:**
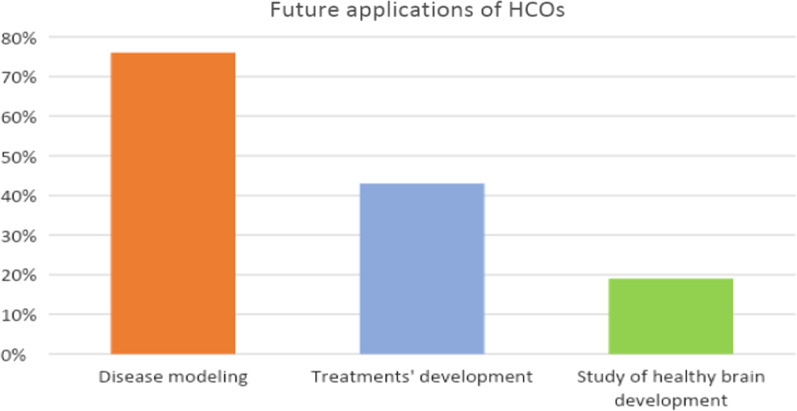
Future applications of HCOs.* Note* Each participant typically mentioned more than one possible future application of HCOs

Most respondents showed a very positive attitude toward this novel biotechnology, particularly with regard to HCOs connected to assembloids. Disease modeling and development of treatment strategies—such as drug screening and cell-replacement therapies—were the most quoted potential applications, followed by using HCOs to understand evolutionary quandaries that cannot be analyzed through animal models (Fig. 1). Some scientists, however, did not embrace this positive framework: some respondents remarked on the high level of hype that such technologies had, despite the lack of certainty about the real translatability of their results or the impacts that they currently have or will have in the future: “At the moment it is a total mess, in my opinion. I think there are too many people publishing too much rubbish, because whatever you do something different happens, so you can create new data very easily and publish them very easily, because, at the moment, almost everything that is written gets published” (P10). Along this line, different respondents showed a critical attitude toward some ambitious claims that compare the activity recorded in HCOs to the activity measured in preterm infants [20].

### Theme 3: personal attitude toward the ethical debate

If most participants were skeptical about the emerging consciousness hypothesis, they still showed a very positive attitude toward the current research and the ethical discussion in general, suggesting that it is necessary for ethics and science to work in parallel and together. But it is worth stressing that many researchers did not even respond to the request to participate in the survey on ethical themes.

Among the respondents, the positive framework, however, was not fully shared: the problem of consciousness was defined by some as “artificial”, and other issues were pointed out as more pertinent, such as proper material recruitment, ownership, standardization of procedures and data sharing, and consent. This last issue—informed consent—requires consideration in and of itself. There is a degree of polarization in scientists’ opinion on whether donors should be specifically informed that their cells can be used to grow HCOs or if a broader, general informed consent is sufficient. Advocates of the specific informed consent argued that it should become a mandatory step, while others claimed that it would not only be unnecessary, but it would also lengthen an already complex process.

The same polarization was found when interviewees were asked about the current guidelines: while some argued that, at the current stage, they are fine, others suggested that regulatory protocols always lag behind the fast pace of research, and therefore one should begin to think about novel ones, specifically thought for HCOs. Interestingly, it seems that the ethical debate is not perceived as threatening by researchers: very few expressed concerns about the possibility of ethical inquiry slowing research down, whereas the majority agreed that this is not going to be the case. Some participants, however, did express the concern that misinformation might have repercussions on research: “I believe that misinformation will affect it [research]. […]. I think that, usually, people are […] scared about things they do not know, or they think are different from what they really are. I think, if we really understand brain organoid research, then we can make informed decisions, but this could be really affected, especially when there are some wrong names, and maybe too much expectation” (P11).

### Theme 4: personal attitude toward the public

As partially anticipated by the divergent opinions on the use of the term “mini-brain”, participants were also asked about what they think about the relationship between science and the general audience, specifically in the framework of organoid research. Again, opposite opinions were found: some scientists argued that this relationship is not satisfactory, because either the scientific community does not know how to communicate to the public, or the public is not interested in keeping updated on scientific findings, or a combination of the two; others, instead, had a very positive opinion of this relationship. Interestingly, it seems that, in the personal experience of the sample considered, the public does not share neuroethicists’ concerns, especially on consciousness.

Table 2 sums up the results, highlighting the frequency of response for each domain and subdomain identifiable within the general themes just presented (Table 3, Additional file 1, includes extensive anonymized quotations from the interviews).

**Table 2:** General, typical, variant, and uncommon subdomains within each theme considered, based on the qualitative analysis of researchers’ stance over the ethical discussion on HCOs

General themes, domains, and sub-domains	Frequency
*On consciousness and moral status*	
Consciousness	
Skepticism about consciousness emerging in HCOs today	General
Skepticism about consciousness emerging in HCOs in the future	Variant
Repercussions on the usage of HCOs if consciousness is detected	Typical
Consciousness in HCOs would be similar to that of flies or grasshoppers	Uncommon
Moral status	
There is no ethically relevant difference between HCOs and other tissue products	Typical
There is an ethically relevant difference between HCOs and other tissue products	Variant
Human/non-human chimeras	
Chimeras are no different compared to other animals without HCOs grafts	General
Personal ethical concerns	
No personal ethical concern	Typical
*Personal experience with HCOs*	
HCOs as “mini-brains”	
HCOs are not “mini-brains”	Typical
Rejection of the usage of the term “mini-brains”	Variant
Acceptance of the usage of the term “mini-brains”	Variant
Effective contribution of HCOs to research	
Disease modeling as future application	Typical
Treatment development as future application	Variant
Study of healthy human brain development as future application	Variant
Skepticism on the effective usefulness of HCOs	Variant
*Personal attitude toward the ethical debate*	
Positive attitude toward the ethical debate	Typical
Informed consent	
Need of specific informed consent for donating for HCOs	Typical
No need of specific informed consent for donating for HCOs	Variant
Guidelines and regulations	
Current guidelines are sufficient	Typical
Current guidelines should be updated	Variant
Impact of ethics over research	
Concerns that the ethical discussion could slow research down	Variant
No concern that the ethical discussion could slow research down	Typical
*Personal attitude toward the public*	
Good relationship between science and public	Variant
Science-public relationship could be improved	Variant
Poor relationship between science and public	Variant

*Note* General = 21–17 participants, typical = 16–10 participants, variant = 9–4 participants, uncommon < 3 participants

## Discussion

A recent study [17] investigated how the ethical and social issues of brain organoids are portrayed in the media. The authors found not only an increased polarization in the media coverage but also a misplaced portrayal of the concerns, where less scientifically-grounded issues—such as the one about consciousness—seem to have a more central role than other, more pertinent matters, like the need for more precise regulatory guidelines. The same misplacement was also recognized by the sample considered here. As interesting and relevant as it may be to think about the possibility of consciousness emerging in a model of the human brain, this concern is not yet considered pertinent by the scientific community. The language used also reflects this skepticism. Some participants defined the entire ethical debate as “not pertinent”, likening HCOs to “something you take out of your nose”.

This word choice is telling: HCOs are not seen as something alive, in the ethically relevant meaning of the term, but rather as akin to other types of cell cultures, like two-dimensional ones. This result stands in contrast to other research findings that considered the perspective of patients and the general public [3, 9]. While these previous studies would suggest that HCOs are somehow perceived to be different compared to other tissue products, according to our sample they are not taken to be morally problematic entities per se. Rather, the relevant ethical issues to be addressed concern the provenance of stem cells and the good management of the material.

The skeptical perspective on HCOs’ potential consciousness affects the other topics analyzed, starting from their moral status. Participants claimed that no specific kind of moral status should be attributed to HCOs, due to their alleged absence of consciousness. Only *if* some form of sentience was detected, participants would agree that novel moral consideration would be necessary. Previous studies [3, 9] had already pointed out that the ethical concerns perceived are correlated to the complexity that HCOs could eventually reach. Koplin and Savulescu [12] proposed a layering of the moral status of HCOs and their corresponding protection in research, depending on their development of consciousness and higher cognitive capacities. It should be noted, however, that while different scholars (e.g., [1, 16]) argue that in cases of uncertainty it is better to err “on the side of generosity and treat them as if they have at least partial moral status” [12], our participants seem to lean on the other side, preferring to be sure of consciousness arising in HCOs before committing to any consideration on their alleged moral status.

Besides being the compass for the discussion on moral status, sentience/consciousness also shaped respondents’ opinions on issues concerning chimeric research. The main ethical issue about human/non-human chimeras is that of cognitive hybridization and it seemed not to concern the respondents. Interviewees maintained that the implanted human cell cultures are neither conscious, nor complex or big enough to make a significant contribution to the non-human animal host; so, the latter cannot become “humanized”.

Participants also seemed generally satisfied with the current research guidelines: as for the discussion on moral status, only the potential emergence of consciousness would require new and stricter rules that would, for instance, prohibit disruptive experimentation or more specifically regulate the usage as brain surrogates. What emerged from scientists’ responses is that current research is not aimed at intentionally developing consciousness in brain models. Accordingly, the ethical relevance of sentience as a by-product is not the same as it would have been if it was the intended goal of lab-grown HCOs or chimeras.

Overall, the ethical orientation of the scientific community seems to remain unconcerned with the issues that many neuroethicists have raised to date. None of the participants reported experiencing any type of personal ethical worry about the research that is being carried out with organoid technology. However, all the respondents declared that they would be prepared to modify their views and behavior if sentience/consciousness were to be found in bigger and more complex HCOs. To borrow a term that was used by some participants, scientists do not wish to become modern “Frankensteins”: if their brain models become able to feel pain, they agree that stricter regulation should be introduced.

Nevertheless, as many participants stressed, the ability to feel pain is not something researchers are unfamiliar with: this is the case for the use of animal models, which certainly experience pain. So, in a similar fashion, new rules could be implemented for HCOs should they turn out to be sentient, in order to better define what is and is not permitted, without entirely prohibiting their use. The possibility of drafting novel guidelines—specifically thought for brain organoids—is seen by some as a natural step further in the employment of these brain proxies in research. In this sense, the lack of discussion in governmental and policy-making contexts [17] is an issue that ought to be addressed promptly. Once again, it should be noted that these are speculative hypotheses: most participants, at the moment, seem to support the current recommendation of the International Society for Stem Cell Research (ISSCR) that exempt HCOs from any specific ethical oversight (International Society for Stem Cell Research, 2021).

Another interesting finding concerns the sharp disagreement that emerged with regard to the informed consent required to get a biological sample from a donor. It seems that at least part of the scientific community is not receptive to the requests coming from potential donors: previous studies, in fact, highlighted that patients and laypeople strongly favor a detailed informed consent [2, 3], some advocating even for the possibility of retaining full decisional authority on the type of research carried out with their cells [9]. If part of our sample seems to be sympathetic to these kinds of requests, others are not. This misalignment between what the scientists are fine with and what the public considers acceptable may become a problematic spot that future research will need to take into consideration.

Finally, participants seemed to be aware of the potential miscommunication of scientific findings to the public and believed that the language used for describing organoids plays an important role. Indeed, there is strong disagreement on whether the use of terms such as “mini-brains” is correct or should be avoided. This result fits with the framework already drafted by previous research [2, 9] that highlighted the pivotal role of language and the imagination in the science-public discourse.

It should be highlighted that almost all participants showed a very positive attitude toward this study. Many respondents stressed the importance of keeping a channel open between scientists and ethicists, where the former are ready to listen to and consider the concerns of the latter, who are willing to leverage on the results coming from science to shape the ethical debate and shift it toward the most delicate outcomes.

Overall, there seems to be some discord between researchers' willingness to consider ethical issues arising from human organoid research and their specific positions on the subject. Central to this is the belief, shared by most researchers, that HCOs cannot become conscious and consequently do not enjoy a significant moral status. In this way, the desired dialogue among the various stakeholders involved in the field does not seem to be easily achieved. Yet, the most recent findings [8] indicate that such dialogue is important and necessary.

In this sense, the present research is offered as a helpful—if initial—contribution to setting up a shared ethical framework between the researchers, the ethicists, and the public. The results seem to testify the need to reach a larger consensus on what is relevant from an ethical point of view. Indeed, as revealed by a comparison of the orientations of the researchers interviewed, the evolving scientific literature, and the various aspects of regulation still not made up to date nor homogeneous, it is important to continue on the path of as broad a convergence as possible in the domain of a shared research ethics.

## Conclusion

The aim of this research was to explore the ethical attitude of researchers working on human cerebral organoids and how the ethical debate affects the scientific community. To the authors’ current knowledge, the present study is the first one to investigate scientists’ perspectives.

Our findings show that there is a widespread skepticism about the emergence of consciousness in HCOs, but there is also a general agreement that the use of HCOs would be impacted if consciousness were detected. Most researchers believe that there are no significant differences in moral status between HCOs and other tissue products. Additionally, the majority of respondents believe that chimeras should be treated no differently than other animals without HCO grafts. However, not all researchers have a positive attitude toward the ethical debate surrounding HCOs, and some do not believe that specific informed consent is necessary for donating tissue for HCO research. Despite this, the majority feel that current guidelines are sufficient and are not concerned that the ethical discussion will impede research progress.

That being said, the following limitations should be taken into consideration. Recruiting participants was a troublesome process: more than three-quarters of the PIs contacted did not show any willingness to talk about the ethics of HCOs. This could have involuntarily created a selection bias, whereby the positive attitude detected in participants’ responses is not indicative of the general sentiment of the scientific community: there might be a strong correlation between the willingness to participate and a positive outlook on the ethical discussion. It is possible that the low level of participation in this study could be due to scientists' belief that there are no ethically relevant issues at stake, particularly regarding consciousness. This reluctance could indicate a need to engage researchers in discussions about ethical issues outside of the laboratory. It should be noted that the small sample size of this study may limit the generalizability of the findings, particularly considering that all participants had a Western cultural background, which could have influenced their responses to questions about moral status and other ethical issues. It is not possible to quantify the extent to which cultural background may have affected these responses.

All in all, it looks like the dialogue between science and ethics, biology and philosophical questions is a fertile ground where new ideas can be seeded and grown. It follows that this project should be seen as a first step toward a body of research aiming at smoothing and cultivating the discussion between science and ethics of research.

## Supplementary Information

**Additional file 1**. Supplementary Material: exemplary quotation by theme.

## Data Availability

Full transcripts of the interviews are not reported here due to the risk of disclosing personal information, but representative quotes are available in “Additional file 1”.

## Abbreviations

HCOs: Human cerebral organoids
iPSC: Induced pluripotent stem cell
ES: Embryonic stem cell
