# Supplementary material for: Human cerebral organoids: the ethical stance of scientists
Source: Stem Cell Res Ther. 2023 Apr 1;14:59. doi: 10.1186/s13287-023-03291-x (PMC10068143; doi:10.1186/s13287-023-03291-x)
Supplement: Supplementary file 1 — Additional file 1. Supplementary Material: exemplary quotation by theme. [file 13287_2023_3291_MOESM1_ESM.pdf]

Supplementary Materials

Table 3

Exemplary Quotations by Theme

Themes	Quotations
Theme 1: On consciousness and moral status	**Q1:** "[…] organoids, in terms of size, in terms of number of neurons probably lay somewhere between cockroaches and flies. In terms of complexity and organization, which is another important part, they are below flies, they are below worms even […] If we just take size, the magnitude of difference to humans, is so huge it is actually really hard for us to grasp it, it is one of those gigantic numbers that is very difficult for human beings to really understand, it is enormous. I calculated it, at one point, and in order to make an organoid that would be that big, I would need a tissue culture dish that is as big as my whole house. It is not going to happen." (P2)
	**Q2**: "[…] of course, the brain without a body is like fish out of water: you cannot have one without the other." (P10)
	**Q3**: "[…] it is hard to measure consciousness, and we do not even know exactly what consciousness is, yet. I guess we would really need to define that, in order to understand whether we should be concerned or not." (P3)
	**Q4**: "I think that still, to me, it is similar to other organoids of other part of the body, liver, pancreatic organoids, because the organization is too immature to expect something more." (P16)
	**Q5**: "Personally, no [I do not have any ethical concern working with human brain organoids]. Not myself, not the people we have at the lab working with them" (P20)
	**Q6**: "[…] the level and the number and the level of integration of these cells is just so basic that is not even having a change in the animal, and if it was to have a change in animal it would definitely not be […] it would be extremely unlikely that we would hear a behavioral change that is like on the direction of becoming humanized. Rather, it will be more likely that they will resolve having some sort of seizures, of like unexpected or asynchronous brain activity." (P22)
	**Q7**: "I do not think it is possible [to have consciousness in organoids]." (P11)
	**Q8**: "What do you think a fly, or a mosquito has consciousness? Yes, or no? I think that it has consciousness as a fly, or as a mosquito, so it does not stop me from, let's say, killing that mosquito if I am beaten. An organoid has consciousness as an organoid, not as a human being." (P21)
	**Q9**: "I think that, if you get there, to this point [somehow there will be a way of assessing pain with certainty, and there are proves that organoids do indeed feel it] and you are sure you are dealing with a system, then you apply the same concerns, procedures, and precautions that you apply to any other model." (P1)

Theme 2: Personal experience with HCOs

Q10: "[…] when you actually work with these things, they look like something you take out of your nose. They are like little blobs; they really do not look like actual brains." (P2)

Q11: "[…] we call 'mini-brain' but, biologically, [it] is not yet a mini-brain." (P14)

Q12: "I think that 'mini-brains' is excellent because I think where the miscommunication occurs is when we call them 'organoids' or 'cerebral organoids', because then the public cannot access something which is relevant to them. I think the more we call them 'mini-brains' the better because it overstates the biology." (P12)

Q13: "[…] we never call brain organoids 'mini-brains', as many other in the field do, because, despite this terminology is very useful to make people that are not in the field understand what actually these three-dimensional models are, this can also be misinterpreted […]." (P11)

Q14: "I know that there has been a discussion in the field and some people claim, or have claimed, that these organoids have activities that are similar to those that are present in new-born human people, human brain. I think this is not a fact, it is an opinion, and the field is debating to what extent this is actually true or not." (P8)

Theme 3: Personal attitude towards the ethical debate

Q15: "I am very thrilled with this conversation; it is something that I have not thought about. I was thinking that I am going to tell my students that I had this conversation with you, and that there are people thinking about that, because they can also think about these things: that is science." (P13)

Q16: "I think this is really, in some level, an artificial topic. Researchers like myself, and others that are way more prominent and visible do a lot of interviews and discussions about that, to make clear what is actually the state of the art and what is fantasy, and, as long as we continue to do that, I think we are in a good shape." (P6)

Q17"[…] I do feel like there is a weakness in terms of the patient's consent to this type of things. These are all anonymized cell lines, and so there would never be a link, but nonetheless I think there are probably people out there who, if they knew that their cells might be used to generate brain organoids, they may or may not want that." (P2)

Q18: "I think at the moment the general consent is that if you give some [cells], then we can do whatever we want with them. To change this, I am not sure if it would be beneficial, because I think it would… People would give a weight to them [inaudible], and they would imagine their cells as a minuscule brain." (P7)

Q19: "As things are now, I do not think we need guidelines, because again, we do not have guidelines for lung organoids, liver organoids, so, as things are now, we do not need, except normal guidelines of where to recover stem cells, and that is it." (P4)

Q20: “No, they [guidelines] are not sufficient. I mean, always. That was what I meant initially that the ethicists need always to follow us a little bit because we are, most likely, driv[ing] the questions for the ethicist. You come up with some research results and ethicists just never thought about that. There are artificial brains. If you would be asked ten years ago, you would say: “Ok, it is a science fiction what we are talking about”. But I believe it is a dynamic process: it should always be reviewed on, let's say, on not on a yearly basis, but every five years it should be reviewed, whether it is still up to the level of the present knowledge.” (P18)

Theme 4: Personal attitude towards the public

Q21: “I think, in general, the general public should be more informed. It is information what probably is missing: only the bad things maybe come and generate the biggest debate of all.” (P5)

Q22: “I have a very, very good opinion on the public’s view on science. […] I have to say that every time I do one of these things [public engagement events], I do take an actual brain organoid with me, to show people what it is that I am talking about, so it might also be, again, what I said about letting people actually see what it is you are talking about, it really does impact the way you view it, from an ethical standpoint. I think that is important: people should really see what it is that they are talking about.” (P2)

Q23: “[…] we have had a really big event here [identifying information] along with 500 lays, who wanted to inform themselves about brain organoids, but, honestly, the questions were different than raising ethical concerns. The questions that the public had were more in the therapeutic potential of the brain organoids, or more technical questions, but there was nobody standing up and expressing his thought that there may be a problem.” (P19)

Q24: “[…] we try to explain in the most simple way that we can, and the response we get usually from patients is a lot of enthusiasm about this. So, in our experience, patients are not scared about the reproduction of their neurons, let's call it that way: they are actually excited about it.” (P17)
